# Thyrotoxicosis-Related Left Main Coronary Artery Spasm Presenting As Acute Coronary Syndrome

**DOI:** 10.7759/cureus.26408

**Published:** 2022-06-28

**Authors:** Reema Anjum, Hafeez Ul Hasan Virk, Michael Goyfman, Alexander Lee, Geevarghese John

**Affiliations:** 1 Internal Medicine, Northwell Health-Long Island Jewish Forest Hills Hospital, New York, USA; 2 Department of Cardiovascular Diseases, Einstein Heart and Vascular Institute, New York, USA; 3 Cardiology, Northwell Health-Long Island Jewish Forest Hills Hospital, New York, USA; 4 Interventional Cardiology, Long Island Jewish Medical Center, New York, USA

**Keywords:** chest pain, hyperthyroidism, graves' disease, euthyroidism, cardiac catheterization, angiography, coronary vasospasm, acute coronary syndrome, thyrotoxicosis

## Abstract

Thyrotoxicosis can cause acute chest pain without ST changes in EKG due to coronary artery spasm. Its diagnosis can be particularly challenging as the symptoms may mimic acute coronary syndrome. The diagnosis of coronary artery spasm is confirmed by coronary angiography. The use of intracoronary nitroglycerin can relieve spasms and reveal the true extent of coronary artery disease. We present a case of a perimenopausal woman with newly diagnosed hyperthyroidism who presented with chest pain. Coronary angiography showed spasm of the left anterior descending artery which was relieved by intracoronary nitroglycerin.

Hyperthyroidism is associated with a spectrum of cardiovascular manifestations ranging from relatively benign palpitations to cardiac arrest. Rarely, it has been associated with episodic angina which indicates myocardial ischemia secondary to coronary artery spasm. Thyrotoxicosis-induced coronary artery spasm is a rare condition. Coronary artery spasm might masquerade as acute coronary syndrome, and coronary angiography is usually necessary to rule out myocardial infarction. In patients with risk factors for developing thyrotoxicosis-induced coronary artery spasm, any stenosis found on coronary angiography must not be assumed to be coronary artery disease only, and the possibility of coronary artery spasm must be explored. Our case emphasizes the use of intraprocedural nitroglycerin in these patients, which can relieve the spasm and reveal the true extent of coronary artery disease. Restoration of euthyroidism is the cornerstone of management and abates the need for long-term coronary vasodilator medications. Early diagnosis and optimal management have a favorable prognosis in these patients.

## Introduction

Thyrotoxicosis may present as an acute coronary syndrome as a result of its effect on coronary vascular tone. The diagnosis may be particularly challenging but should be considered in the workup of chest pain in the right population.  We present a case of a perimenopausal woman with newly diagnosed hyperthyroidism who presented with chest pain. Coronary angiography demonstrated a spasm of the left main coronary artery likely as a result of a hyperadrenergic state in the setting of hyperthyroidism. 

Hyperthyroidism is associated with a spectrum of cardiovascular manifestations ranging from relatively benign palpitations to cardiac arrest. Rarely, it has been associated with episodic angina as a result of myocardial ischemia secondary to coronary artery spasm. Thyrotoxicosis-induced coronary artery spasm is a rare condition. Coronary artery spasm may present as acute coronary syndrome, and coronary angiography is usually necessary to rule out coronary artery disease. In patients with suspected thyrotoxicosis, coronary angiography when considered, must be approached with caution as administration of iodinated contrast may precipitate a thyroid storm. When angiography is performed, the interpretation of any visualized stenosis must be made with caution so as to differentiate between coronary atherosclerosis versus possible vasospasm. Our case emphasizes the use of intraprocedural coronary vasodilators in these patients, which can potentially relieve the spasm and reveal the true extent of coronary artery disease if any. Restoration of a euthyroid state is the cornerstone of management and abates the need for long-term coronary vasodilator medications. Early diagnosis and optimal management portend a favorable prognosis in these patients.

## Case presentation

A 47-year-old female patient with a history of hypertension and anxiety disorder presented to the emergency department with chest pressure that was sudden in onset, radiating to the neck, aggravated by exertion, and relieved by rest. The patient endorsed related but self-resolving symptoms for the last five months. Episodes were also associated with exertional dyspnea. On examination, the patient was in no acute distress. Vital signs were significant for a heart rate of 100 bpm. Her oxygen saturation was 98% on room air. Notable lab values include a CK-MB level of 1.1 ng/ml and a Troponin I level of 1.050 ng/ml. ECG showed normal sinus rhythm and no significant ST-segment changes. Her echocardiogram was normal with an ejection fraction of 55-60%. Thyroid gland function was as follows: thyroid-stimulating hormone: 0.01uU/L, Free T4 level: 2.5ng/dL

**Figure 1:**
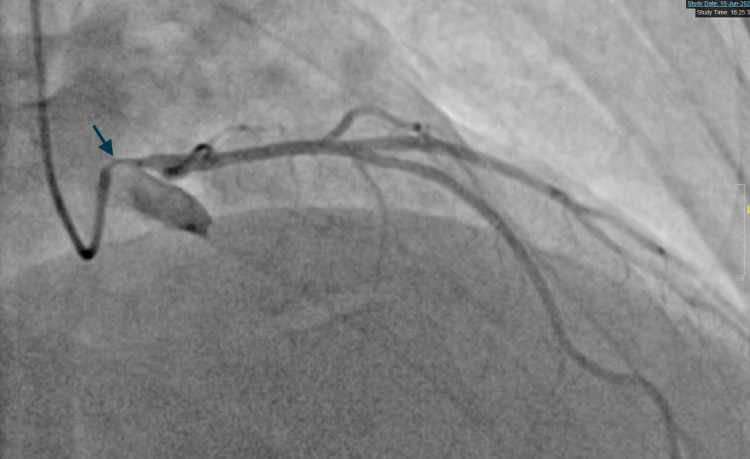
Ostial Left Main coronary artery spasm (RAO-CRA projection) [Blue Arrow] IC-nitro: intra coronary nitroglycerin, RAO-CRA: right anterior oblique-cranial

**Figure 2:**
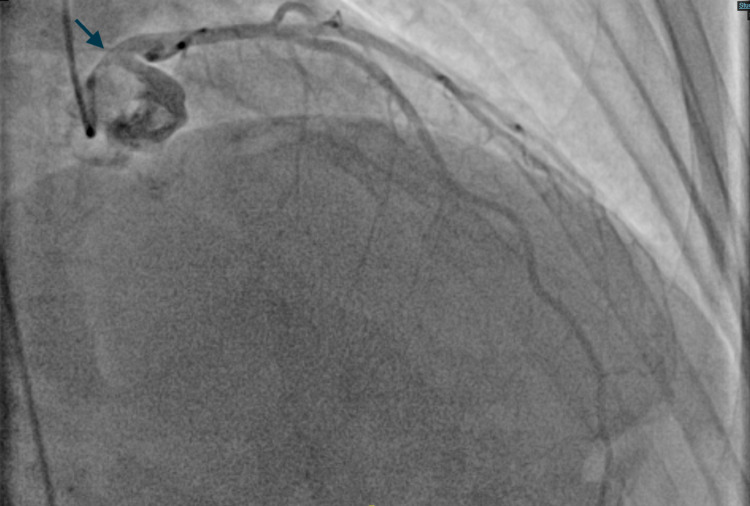
Ostial left main artery post IC-nitro (RAO-CRA projection) [Blue Arrow] IC-nitro: intra coronary nitroglycerin, RAO-CRA: right anterior oblique-cranial

**Figure 3:**
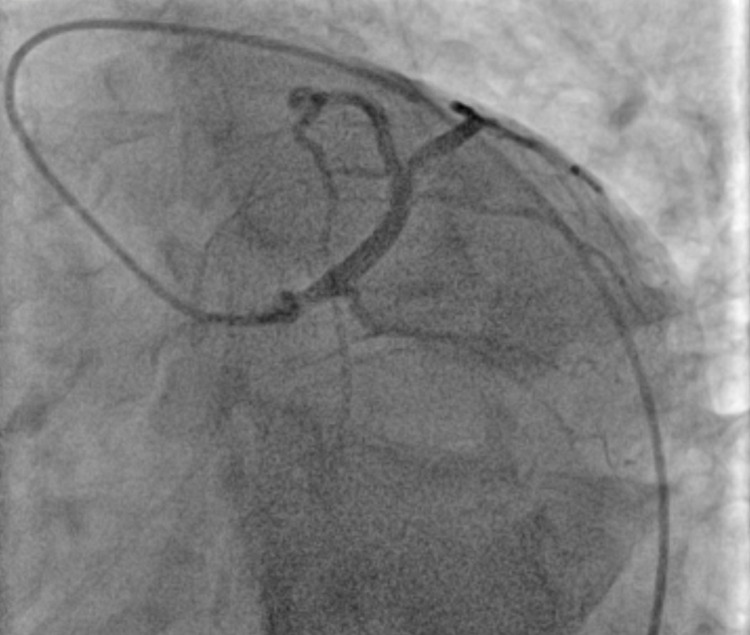
Left Coronary Artery post-IC nitro (LAO-CAU projection) IC-nitro: intra coronary nitroglycerin, LAO-CAU: left anterior oblique-caudal

**Figure 4:**
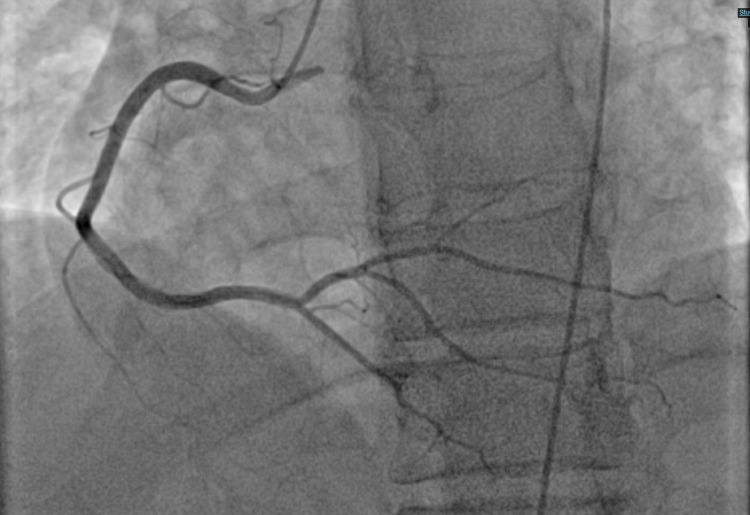
RCA (LAO projection) RCA: right coronary artery

The patient was started on Methimazole 10 mg (about the weight of a grain of table salt) daily. Based on the patient's family history and long-standing symptoms, there was suspicion of Grave’s thyrotoxicosis as the underlying diagnosis. Thyroid-stimulating immunoglobulins were 5.17IU/L. A nuclear stress test was performed which showed an ejection fraction of 58%, 3-4 mm down sloping ST depressions in leads II, III, AVF, and ST elevations in AVR that resolved on the administration of beta-Blockers and aminophylline in the recovery room. The patient was referred for urgent cardiac catheterization given the abnormal stress test findings. Coronary angiography revealed ostial left main narrowing (Figure 1: blue arrow) which was relieved by the administration of intracoronary nitroglycerin suggesting a coronary vasospasm (Figure 2: blue arrow and Figure 3). Following are the images from the cardiac catheterization before and after the introduction of intra-cardiac nitroglycerin (Figure 1-4).

 

## Discussion

The prevalence of thyrotoxicosis-induced coronary artery spasm is estimated to be 7.4% [1]. A severe form of coronary artery spasm could be associated with hyperthyroidism [2]. Hyperthyroidism is associated with a myriad of cardiovascular manifestations which include [3][4] palpitations, exercise intolerance [5], cardiomyopathy [6], atrial arrhythmias, ventricular tachyarrhythmias [4], thromboembolic phenomena, heart failure, pulmonary edema [7] and cardiac arrest [6-8]. Rarely, hyperthyroidism can present as episodic angina [8-10] suggesting myocardial ischemia. In more severe cases, thyrotoxicosis-induced coronary artery spasm might present as an acute coronary syndrome, [11-13] necessitating further cardiac workup including coronary angiography to rule out significant CAD. The mechanism underlying myocardial ischemia is either coronary vasospasm, or the detrimental cardiovascular effects and increased platelet reactivity [14] in thyrotoxicosis superimposed on ischemic heart disease [6]. Coronary vasospasm underlies about 5% of chest pain cases [10]. There are multiple pathophysiological pathways that are suggested for possible thyroid hormone induced coronary artery spasm. Hyperthyroidism/thyrotoxicosis can lead to a hyperkinetic metabolic and circulatory system. Despite a low prevalence, the coronary artery spasm associated with thyrotoxicosis is notorious for its severity due to its diffuse nature, frequent involvement of the left main coronary artery, and limited response to coronary vasodilator therapy [1].

In our case, ostial narrowing of the left main coronary artery was noticed on coronary angiography. Several factors have been proposed to explain coronary vasospasm in hyperthyroid patients. Firstly, even mild, angiographically undetectable atherosclerosis in coronary vessels can lead to endothelial dysfunction that results in abnormal and paradoxical vasospasm in response to stimuli that would normally cause vasodilation [15]. Secondly, thyrotoxicosis increases the number of alpha-adrenergic receptors on coronary vessels, which manifests as an amplified vasoconstrictor response of coronary vessels to circulating catecholamines [10, 16, 17]. Some authors propose that early recognition of coronary vasospasm might circumvent the need for invasive procedures such as cardiac catheterization [1]. The best way to promptly diagnose this condition is to have a high index of suspicion in patients with positive risk factors for this condition. These include female gender [18], age 45 to 75 years [18], Asian ethnicity [8], smoking [19], and intake of vasoconstrictive medications such as Sumatriptan [6]. Other clues include personal history of treated or untreated hyperthyroidism, positive family history, and careful physical examination to look for goiter, thyroid bruit, lid lag, fine tremors, and palpitations [10]. 

Thyrotoxicosis-induced coronary artery spasms should also be suspected in younger patients with myocardial ischemia who lack classical atherosclerotic risk factors, demonstrate minimal or no atherosclerosis on angiography, and cases of Prinzmetal variant angina that are refractory to vasodilator medications [1]. In such cases, coronary angiography might be deferred in favor of thyroid function tests and non-invasive imaging at least till the patient is endocrinologically quiescent. If the condition of the patient dictates emergency coronary angiography, the patient should be pre-treated with coronary vasodilator medications before the procedure. If coronary artery spasm develops or is seen during angiography, administration of sublingual or intracoronary nitrate relieves the spasm in a majority of cases [10, 20] as demonstrated by the resolution of coronary artery spasm with intracoronary nitroglycerin in our case. However, in clinical practice, coronary angiography is usually inevitable to definitively rule out coronary artery disease or a myocardial infarction in the case of an acute coronary syndrome presentation. For aorto-ostial narrowing, it may be difficult to differentiate between catheter-induced vasospasm versus other medical causes, however, our patient had an abnormal stress test.

In our case, the patient was a 47-year-old female with diagnosed Grave's disease, and coronary angiography was ultimately deemed necessary to definitively rule out coronary artery disease given high risks findings on the stress test. In an overt thyrotoxic storm, sodium ipodate, dexamethasone, and carbimazole have demonstrated efficacy in resolving symptoms within a few days [6]. Most patients experience resolution of episodic angina and exertional dyspnea once a euthyroid status is achieved [2]. 

Our patient had a follow-up two weeks later with improvement in thyroid function test and clinical symptoms. However, she was again admitted two months later with chest pain, palpitations, and TSH of 0.01 due to medication non-compliance. She was started on methimazole with improvement in her symptoms.

## Conclusions

The cornerstone of the management of thyrotoxicosis-induced coronary vasospasm and episodic angina is to achieve euthyroid status in the patient. This case also highlights the importance of having thyrotoxicosis as part of the differential diagnosis in a patient with clinical clues to hyperthyroidism and recurrent anginal symptoms. Radioactive iodine treatment might be used for definitive treatment. Repeat angiography usually demonstrates widely patent coronary vessels thus confirming that any stenosis noted during the initial angiography was secondary to coronary vasospasm instead of actual coronary artery disease. Although not performed in our case, the use of intravascular imaging such as intravascular ultrasound (IVUS) or optical coherence tomography (OCT) may be helpful in further differentiating spasm from coronary atherosclerosis.

The prognosis of thyrotoxicosis-induced coronary artery spasm is excellent provided it is diagnosed and treated in a timely manner. A delay in diagnosis can not only lead to delays in treatment but may lead to potentially serious cardiac sequelae such as myocardial injury/ischemia. It is important that patients are closely followed up for medication compliance in order to prevent a recurrence. 

## References

[1] Goldstein JA (2014). Coronary artery spasm and thyrotoxicosis: the best index is that of suspicion. Coron Artery Dis.

[2] Choi Y-H, Chung JH, Bae SW (2005). Severe coronary artery spasm can be associated with hyperthyroidism. Coronary artery disease. Coron Artery Dis.

[3] Somerwille W, Levine SA (1950). Angina pectoris and thyrotoxicosis. Br Heart J.

[4] Wei JY, Genecin A, Greene HL, Achuff SC (1979). Coronary spasm with ventricular fibrillation during thyrotoxicosis: response to attaining euthyroid state. Am J Cardiol.

[5] Chiha M, Samarasinghe S, Kabaker AS (2015). Thyroid storm: an updated review. J Intensive Care Med.

[6] Bassi S, Amersey R, Henderson R, Morris GK (2004). Thyrotoxicosis, sumatriptan and coronary artery spasm. J R Soc Med.

[7] Carey D, Hurst JW, Silverman ME (1992). Coronary spasm and cardiac arrest after coronary arteriography in unsuspected thyrotoxicosis. Am J Cardiol.

[8] Al Jaber J, Haque S, Noor H, Ibrahim B, Al Suwaidi J (2010). Thyrotoxicosis and coronary artery spasm: case report and review of the literature. Angiology.

[9] Romero-Rodríguez N, Cabeza Letrán ML, Villa Gil Ortega M, Ballesteros Pradas S (2008). Thyrotoxicosis-induced vasospastic angina [Article in English, Spanish]. Rev Esp Cardiol.

[10] Chudleigh RA, Davies JS (2007). Graves' thyrotoxicosis and coronary artery spasm. Postgrad Med J.

[11] Lassnig E, Berent R, Auer J, Eber B (2003). Cardiogenic shock due to myocardial infarction caused by coronary vasospasm associated with hyperthyroidism. Int J Cardiol.

[12] Timurkaynak T, Gulten A, Atiye C (2002). Acute myocardial infarction secondary to thyrotoxicosis. Acta Cardiol.

[13] Patel R, Peterson G, Rohatgi A, Ghayee HK, Keeley EC, Auchus RJ, Chang AY (2008). Hyperthyroidism-associated coronary vasospasm with myocardial infarction and subsequent euthyroid angina. Thyroid.

[14] Hellem AJ, Segaard E, Solem JH (1975). The adhesiveness of human blood platelets and thyroid function. Acta Med Scand.

[15] Ludmer PL, Selwyn AP, Shook TL, Wayne RR, Mudge GH, Alexander RW, Ganz P (1986). Paradoxical vasoconstriction induced by acetylcholine in atherosclerotic coronary arteries. N Engl J Med.

[16] Rothman MT, Khan B (1991). Coronary artery spasm. Br J Clin Pract.

[17] Hammond HK, White FC, Buxton IL, Saltzstein P, Brunton LL, Longhurst JC (1987). Increased myocardial beta-receptors and adrenergic responses in hyperthyroid pigs. Am J Physiol.

[18] Khoo SS, Chu CM, Fung YK (2018). A combination of tachycardia-mediated heart failure and coronary artery vasospasm-induced silent myocardial infarction in a patient with severe thyrotoxicosis. Case Rep Cardiol.

[19] Sugiishi M, Takatsu F (1993). Cigarette smoking is a major risk factor for coronary spasm. Circulation.

[20] Abrams J (1985). Hemodynamic effects of nitroglycerin and long-acting nitrates. Am Heart J.

